# FIXING JEJUNAL MANEUVER TO PREVENT PETERSEN HERNIA IN GASTRIC BYPASS

**DOI:** 10.1590/S0102-6720201500S100019

**Published:** 2015-12

**Authors:** Abdon José MURAD-JUNIOR, Christian Lamar SCHEIBE, Giuliano Peixoto CAMPELO, Roclides Castro de LIMA, Lucianne Maria Moraes Rêgo Pereira MURAD, Eduardo Pachu Raia dos SANTOS, Almino Cardoso RAMOS, José Aparecido VALADÃO

**Affiliations:** 1 São Domingos Hospital, São Luís, MA; 2 Uniceuma, São Luis, MA; 3 Hospital das Clinicas, Federal University of Pernambuco, Recife, PE; 4 Gastro-Obese-Center, São Paulo, SP, Brazil

**Keywords:** Obesity, Bariatric surgery, Surgical technique, Gastric bypass

## Abstract

**Background:**

: Among Roux-en-Y gastric bypass complications is the occurrence of intestinal obstruction by the appearance of internal hernias, which may occur in Petersen space or the opening in mesenteric enteroenteroanastomosis.

**Aim:**

: To evaluate the efficiency and safety in performing a fixing jejunal maneuver in the transverse mesocolon to prevent internal hernia formation in Petersen space.

**Method:**

: Two surgical points between the jejunum and the transverse mesocolon, being 5 cm and 10 cm from duodenojejunal angle are made. In all patients was left Petersen space open and closing the opening of the mesenteric enteroenteroanastomosis.

**Results:**

: Among 52 operated patients, 35 were women (67.3%). The age ranged 18-63 years, mean 39.2 years. BMI ranged from 35 to 56 kg/m^2^ (mean 40.5 kg/m^2^). Mean follow-up was 15.1 months (12-18 months). The operative time ranged from 68-138 min. There were no intraoperative complications, and there were no major postoperative complications and no reoperations. The hospital stay ranged from 2-3 days. During the follow-up, no one patient developed suspect clinical presentation of internal hernia. Follow-up in nine patients (17.3%) showed asymptomatic cholelithiasis and underwent elective laparoscopic cholecystectomy. During these procedures were verified the Petersen space and jejunal fixation. In all nine, there was no herniation of the jejunum to the right side in Petersen space.

**Conclusion:**

: The fixation of the first part of the jejunum to left side of the transverse mesocolon is safe and effective to prevent internal Petersen hernia in RYGB postoperatively in the short and medium term. It may be interesting alternative to closing the Petersen space.

## Abstract (pt)

***Racional*:**

Entre outras complicações do bypass gástrico em Y-de-Roux está a ocorrência de obstrução intestinal pelo aparecimento de hérnias internas, que podem ocorrer no espaço de Petersen ou na abertura mesentérica da enteroenteroanastomose.

**Objetivo:**

: Avaliar a eficiência e a segurança da realização de uma manobra de fixação do jejuno no mesocólon transverso para evitar formação de hérnia interna no espaço de Petersen.

**Método:**

: Realizam-se dois pontos de fixação entre o jejuno e o mesocólon transverso, sendo um a 5 cm e outro a 10 cm do ângulo duodenojejunal. Em todos os pacientes foi deixado o espaço de Petersen aberto e realizado o fechamento da abertura mesentérica da enteroenteroanastomose.

**Resultados:**

: Entre 52 pacientes estudados, 35 eram do sexo feminino (67,3%). A idade variou de 18 a 63 anos, com média de 39,2 anos. O IMC variou de 35 a 56 kg/m^2^ (média de 40,5 kg/m^2^). O seguimento médio foi de 15,1 meses (de 12 a 18 meses). O tempo operatório variou de 68-138 min. Não ocorreram intercorrências intra-operatórias, assim como não houve complicações pós-operatórias maiores e nem reoperações. O período de internação hospitalar variou de 2-3 dias. Durante o seguimento, nenhum paciente desenvolveu quadro suspeito de hérnia interna. Neste período, nove pacientes (17,3%) apresentaram quadro de colecistolitíase (por microcálculos) assintomática, e foram submetidos à colecistectomia videolaparoscópica eletiva. Durante estes procedimentos foram verificados o espaço de Petersen e a fixação jejunal. Em todos os nove, não havia herniação do jejuno para o lado direito do espaço de Petersen.

**Conclusão:**

: A fixação da primeira parte do jejuno ao lado esquerdo do mesocólon transverso é segura e eficiente para evitar hérnia interna de Petersen em pós-operatório de BGYR no curto e médio prazo. Pode ser interessante alternativa ao fechamento do espaço de Petersen.

## INTRODUCTION

The clinical treatment of morbid obesity through lifestyle changes and medications has high failure rates, with only 1-2% of patients showing good results^21^. Bariatric surgery has far superior results than medical treatment^6^. The two most widely used techniques are the vertical gastrectomy (GV) and Roux-en-Y gastric bypass (RYGB). Although the numbers of GV have increased a lot in the last five years and it became the most common bariatric surgery in North America, the RYGB is still the bariatric surgery most performed worldwide^8^. Among other complications, this technique is associated with the occurrence of intestinal obstruction by the appearance of internal hernias, which may occur in Petersen space or in the mesenteric opening of enteroenteroanastomosis^3^^,^ 9. The prevalence of internal hernias varies from 1.3 to 9%^2^^,^ 7 ^,^ 11 and up to 70% are in Petersen space^2^^,^ 3 ^,^ 7.

Because of gastroenterostomy, mainly in antecolic way, there is formation of a space between the mesentery of the alimentary limb and the transverse mesocolon, known as Petersen space, which is the most frequent site of gastric post-bypass internal hernia occurrence. This space after bariatric surgery is considered with this denomination much more by analogy and not representing the originally site described by Whalter Petersen. In 1900, this german surgeon described three cases of internal hernias after operations with reconstruction by a loop gastroenterostomy. The three progressed to death from intestinal obstruction after BII reconstruction where the afferent loop ran after the efferent limb in the space between the mesentery, stomach and colon^16^. In RYGB the formation of internal herniation through this space occurs when the jejunum slides, through duodenojejunal angle, from the left side of the Petersen space to the right, leading to obstruction of biliopancreatic limb, and sometimes also the alimentary limb and common limb when there is invagination of long intestinal segment^18^.

Although not all publications are able to show significant reduction of occurrence of internal hernia with the closure of the Petersen space and of the mesenteric opening left by entero-enteroanastomosis^3^^,^ 19, most studies show that there is a reduction in the incidence of internal hernia when these spaces are closed during the course of BGYR^5^^,^ 15. The closure of the mesenteric opening of the entero-enteroanastomosis is technically easy and when it is performed, it is associated with minimal incidence of internal hernias at this point^22^. Closing the Petersen space during the gastroplasty, on the other hand, is more challenging to the surgeon and can be technically very difficult in some patients, especially in superobese and the ones who have a severe grade of visceral obesity. This closure can be also associated with complications such as bleeding, vascular lesions and hematomas^1^^,^ 13, and despite decreasing the chance of occurrence of internal hernia, it did not totally prevent it; with Petersen hernia may occur in up to 3.8% of patients undergoing gastric bypass with closure of the space during operation^15^.

Understanding the mechanisms of formation of internal hernia in Petersen space, it is possible that an attachment suture from the beginning of the jejunum on the left space side can prevent bowel migration to the right side, avoiding the formation of internal hernia, even keeping Petersen space opened. It would be important to prove safety and efficiency of jejunal fixing maneuver to prevent internal hernia, because it would represent an interesting, quick, and easy to perform alternative to the routine closure of the space.

The aim of this study was to evaluate the efficiency and safety in performing a jejunal fixation in the transverse mesocolon to prevent internal hernia formation within Petersen space after RYGB.

## METHOD

Between January and July 2014, 52 patients of Bariatric and Metabolic Service of São Domingos Hospital, in São Luís, MA, Brazil underwent to laparoscopic RYGB with antecolic reconstruction, and during the procedure it was performed a new technical proposal to fix jejunum segment on the left side of the transverse mesocolon.

**FIGURE 1:**
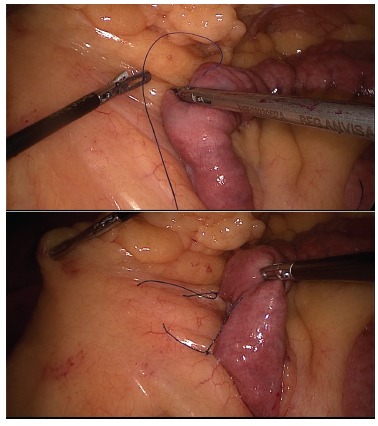
- Technical moments of jejunum fixation to the mesocolon

For measuring of the length of the biliopancreatic limb, transverse mesocolon was moved cranially to identify the duodenojejunal angle. At this time, before measuring the biliopancreatic limb, patients underwent surgical procedure for fixing the beginning of the jejunum in the transverse mesocolon, with deep suture (in an attempt to lessen the chance of undoing the fixation) on ​​the left side of the mesocolon. Initially, fixation was performed with only one stitche with nonabsorbable wire between the jejunum (10 cm from duodenojejunal angle) and the transverse mesocolon. Subsequently, the maneuver has been changed, passing to two stitche between the jejunum and the transverse mesocolon, being 5 cm and 10 cm from the duodenojejunal angle. This modification aimed to avoid the space created between this angle and the 10 cm position stitche, and to reforce the attachment with a second stitche, lessening the chance it could undo the fixation (Figure 1). In all patients Petersen space was left open and the mesenteric opening of the enteroenteroanastomosis was closed. 

To evaluate the safety of the fixing maneuver, hospital stay was evaluated, as well as peri-operative complications, such as abdominal bleeding, hematomas and intestinal obstruction.

The follow-up ranged from 12 to 18 months. During routine assessments (1, 3, 6, 12 and 18 months after surgery) it was asked to the patients if they presented significant abdominal pain, postprandial pain, vomiting or abdominal distension. If they were showing any of these symptoms, they would undergo to total abdominal computed tomography for internal hernia searching.

If there was a need to perform a surgical procedure (unrelated to internal hernia) in these patients, the Petersen space would be explored to evaluate possible asymptomatic internal hernia, and to explore the effectiveness of the fixing manouver, a traction of biliopancreatic limb to the right side of Petersen space would be performed.

## RESULTS

Among 52 patients operated, 35 were women (67.3%). The age ranged 18-63 years, mean 39.2 years. BMI ranged from 35 to 56 kg/m^2^ (mean 40,5 kg/m^2^). Mean follow-up was 15.1 months (12-18 months).

The operative time ranged from 68-138 min, with a mean of 89. There were no intraoperative complications, and there were no major postoperative complications or reoperations. The hospital stay ranged from 2-3 days (50 patients remained in the hospital for two days).

During the quiz in routine evaluations, all patients denied the occurrence of abdominal pain, postprandial pain, vomiting or abdominal distension. There was no need to undergo CT scan or laparoscopy for internal hernia search in any patient. Therefore, no patient presented suspect clinical presentation of internal hernia during this follow-up.

**FIGURE 2:**
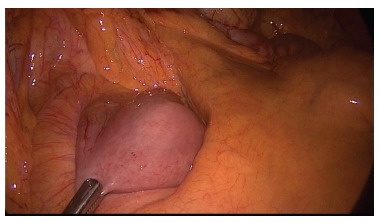
- Late follow-up aspect of the limb attached to the mesocolon

In the same follow-up period, nine patients (17.3%) presented asymptomatic cholelithiasis (small gallstones) and were submitted to elective laparoscopic cholecystectomy. During these procedures it was explored the Petersen space and jejunal fixation. In all nine patients, there was no herniation of the jejunum to the right side Petersen space. Attempts were made to pull the jejunum (biliopancreatic limb) to the right side of the Petersen space; however, the displacement of the intestine was not possible because the jejunum was fixed to the left side of the mesocolon, showing that the fixation was working (Figure 2).

## DISCUSSION

Internal hernia is one of the most common complications following medium and long term of BGYR^13^. Initial symptoms are related to the displacement of the intestine by the hernia hole leading to partial or complete obstruction of the intestine with clinical intermittent abdominal pain, sometimes occurring for months^1^. The typical presentation is mesogastric, postprandial and recurrent pain, radiating to the back. Nausea may be present, but vomiting is rare. Often patients report antalgic position bending the body forward and embracing the abdomen. Diagnosis is not easy, especially for professionals who are not accustomed to monitoring bariatric patients. When the diagnosis is made early, the treatment is effective and safe in general, based on surgical revision, preferably by laparoscopy, which is to reduce the hernia with placement of the intestine in its correct position and to close the defect^4^. But with late diagnosis, strangulation is often, and can lead to large bowel resections or even need bowel transplant, situations involving high mortality^1^^,^ 17. This presentation is even more dramatic when it occurs in special situations, such as during pregnancy^10^^,^ 14.

Not closing the Petersen space and not performing any alternative maneuver leads to an uncomfortable and risky rate of occurrence of internal hernias, ranging from 3-11% in most publications^1^^,^ 15. Higa et al reported a 16% incidence of internal hernias, although not specified how many of these were in Petersen space^11^.

Most publications show lower incidence of Petersen hernia with routine closure of the space while performing the gastric bypass. However, the closure of this space is often technically difficult and time consuming, and many surgeons consider this the most laborious operation time. Routine closure of Petersen space can be associated with complications, and yet reduces, but does not prevent the occurrence of internal hernias^20^. Closing Petersen space can offer opportunity to intestinal slip through small holes leading to strangulation and intestinal necrosis.

In a review article, Kristensen and colleagues reported incidence up to 4,6% of complications related to closure of mesenteric openings, such as hematomas, bleeding and intestinal obstruction^13^. These authors also showed that even with the closure of these openings, there was 1.4% of occurrence of internal hernias. Himpens and colleagues reported incidence of 9.3% of internal hernias despite closing the Petersen space and the enteroenteroanastomosis mesenteric opening, although it has not specified how many of these hernias were in Petersen space^12^. Other studies showed incidence of 1-3,8% of interna hernia in Petersen space, even with the previous closure of the space during the RYGB^13^^,^ 20. In the present study, there was no case of internal hernia, suggesting proper efficiency of the fixation manouver, although with no long follow-up as in other publications.

In the present study the fixation was made quickly and with technical ease in all patients, even in superobese and in patients with severe grade of visceral fat, conditions that hinder the proper closure of Petersen space. Possibly another advantage of the fixing manouver in relation to the closure of Petersen space is related to the case of failures consequences. There would be no serious consequences in the event of the attachment stitches disengage from the mesocolon, while the inadequate closure of the Petersen space, or its partial opening, appear to be associated with more severe herniation, most likely to ischemia and intestinal necrosis due to reduced space to be in.

Although this study reveals promising results of this new technique to prevent internal hernia formation in Petersen space, more studies with larger numbers of patients and longer follow-up are needed to prove the effectiveness in preventing internal hernia in the long term. Randomized prospective studies comparing the efficiency, surgical time, and security between the routine closure of Petersen space and the jejunal fixation manouver shall be encouraged.

## CONCLUSION

The fixation of the first part of the jejunum on left side of the transverse mesocolon is safe and effective to prevent internal hernia in Petersen space in RYGB postoperative in short and medium term. It may be interesting alternative to the closure of the Petersen space.
